# Cancer Therapy-Induced Cardiotoxicity—A Metabolic Perspective on Pathogenesis, Diagnosis and Therapy

**DOI:** 10.3390/ijms23010441

**Published:** 2021-12-31

**Authors:** Anurag Choksey, Kerstin N. Timm

**Affiliations:** 1 Somerville College, University of Oxford, Woodstock Road, Oxford OX2 6HD, UK; anurag.choksey@some.ox.ac.uk; 2 Department of Pharmacology, University of Oxford, Mansfield Road, Oxford OX1 3QT, UK

**Keywords:** cardiotoxicity, metabolism, chemotherapy, heart failure, cardioprotection

## Abstract

Long-term cardiovascular complications of cancer therapy are becoming ever more prevalent due to increased numbers of cancer survivors. Cancer therapy-induced cardiotoxicity (CTIC) is an incompletely understood consequence of various chemotherapies, targeted anti-cancer agents and radiation therapy. It is typically detected clinically by a reduction in cardiac left ventricular ejection fraction, assessed by echocardiography. However, once cardiac functional decline is apparent, this indicates irreversible cardiac damage, highlighting a need for the development of diagnostics which can detect CTIC prior to the onset of functional decline. There is increasing evidence to suggest that pathological alterations to cardiac metabolism play a crucial role in the development of CTIC. This review discusses the metabolic alterations and mechanisms which occur in the development of CTIC, with a focus on doxorubicin, trastuzumab, imatinib, ponatinib, sunitinib and radiotherapy. Potential methods to diagnose and predict CTIC prior to functional cardiac decline in the clinic are evaluated, with a view to both biomarker and imaging-based approaches. Finally, the therapeutic potential of therapies which manipulate cardiac metabolism in the context of adjuvant cardioprotection against CTIC is examined. Together, an integrated view of the role of metabolism in pathogenesis, diagnosis and treatment is presented.

## 1. Introduction

Globally, the incidence of cancer is on the rise, and this trend is expected to continue, in part due to population aging [1]. Yet, thanks to large steps forward in cancer therapy, in many countries mortality rates are now falling [1]. This has created a growing population of cancer survivors, who face the long-term complications of cancer therapy. Cardiotoxicity, referring to cardiac damage and dysfunction, is a severe cause of co-morbidity and mortality in cancer therapies, with the range of therapies associated with cardiotoxicity including: doxorubicin, trastuzumab, sunitinib, imatinib, ponatinib and radiotherapy [2]. Despite extensive research, the pathophysiology of cancer therapy-induced cardiotoxicity (CTIC) with these agents remains unclear. Further, there remains a clinically unmet need to identify which patients are at risk of developing CTIC, especially heart failure. In this regard, imaging techniques and biomarkers are needed to detect at-risk patients before cardiac functional decline becomes apparent. Finally, there is a requirement for more targeted therapeutic options in these patients, which are currently limited to the standard of care heart failure medication such as angiotensin-converting enzyme inhibitors and beta blockers [3].

As a mechanical pump, the heart must constantly generate ATP (adenosine triphosphate) to sustain continuous contraction [4]. This ATP is rapidly hydrolysed to fuel both contractile shortening and ion pumps, such as sarcoplasmic reticulum Ca^2+^-ATPase, amongst others. Normally, the main metabolic substrates of the heart are fatty acids (60–90%) and carbohydrates (10–40%), with more than 95% of the ATP in the heart being generated through oxidative phosphorylation, and a small proportion through glycolysis, yielding lactate. With a high turnover of ATP, there is fine matching of ATP hydrolysis and ATP generation in order to maintain contractile function, requiring exquisite metabolic control to meet cardiac demand [4]. A loss of this cardiac metabolic control can be seen in ischaemic heart failure [5], and alterations to cardiac metabolism likely play a role in the pathophysiology of CTIC [6,7]. In this review, we will focus on the long-term consequences of several cancer therapies, namely, their role in causing heart failure. We will discuss their effects on cardiac metabolism and explore how dysregulated metabolism can be used both for detection and as drug targets in CTIC. We shall explore doxorubicin, an anthracycline chemotherapeutic; trastuzumab, a HER2 inhibitor; the tyrosine kinase inhibitor sunitinib, imatinib and ponatinib, as well as radiotherapy.

Clinically, should metabolic changes occur prior to functional deficits within the myocardium, an early detection of metabolic shifts could provide a sensitive, predictive test of CTIC. There has been rapid development in this field, and here we shall explore the feasibility of using blood biomarkers, ^18^F-fluorodeoxyglucose PET/CT (^18^F-FDG PET/CT) and hyperpolarised ^13^C magnetic resonance imaging (MRI) to detect early alterations in metabolism, preceding CTIC. Whilst early detection of CTIC could allow for rapid cessation of cancer therapy in affected patients to minimise further damage, this would affect cancer outcomes. Instead, the development of adjuvant cardioprotective therapies for delivery to CTIC-vulnerable patients could revolutionise their healthcare, meaning effective cancer therapies could continue to be used, whilst also preventing myocardial damage. As altered cardiac metabolism may represent a pathogenic cause of CTIC, agents which act upon myocardial metabolism could prevent these changes and provide a cardioprotective solution. In this review, we shall examine the roles of metformin, the SGLT2 inhibitors and resveratrol to determine their potential as future adjuvant therapies for cancer patients on cardiotoxic chemo- or radiotherapy.

## 2. Metabolic Dysfunction Leading to Cardiotoxicity of Specific Chemotherapies

### 2.1. Doxorubicin

**Figure 1:**
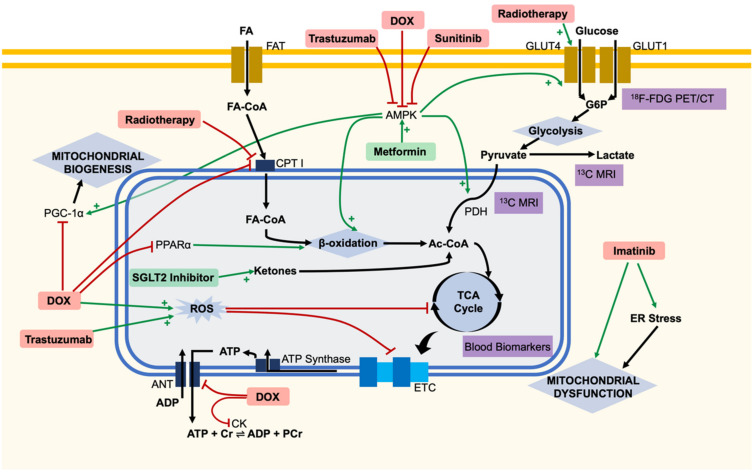
Overview of metabolic changes within the cardiomyocyte in the pathogenesis of cancer therapy-induced cardiotoxicity. Cartoon representation of some explored targets of cancer therapies, with potential diagnostic techniques and therapeutics highlighted. Doxorubicin (DOX) has been linked to widespread metabolic dysfunction throughout the cell. Doxorubicin, trastuzumab and sunitinib have all been linked to the inhibition of adenosine monophosphate-activated protein kinase (AMPK), leading to downstream metabolic dysfunction. Glucose uptake into cells can be detected by ^18^F-fluorodeoxyglucose (^18^F-FDG) positron emission tomography/computed tomography (PET/CT) imaging clinically. Circulating TCA cycle metabolites have been detected in patients and proposed as blood biomarkers. ^13^C hyperpolarised magnetic resonance imaging (MRI) has been used to measure the flux through PDH, both preclinically and in patients. Metformin may provide cardioprotection through activation of AMPK. The sodium-glucose cotransporter 2 (SGLT2) inhibitors may provide an alternative cardiac substrate of ketones to avoid bioenergetic failure. Ac-CoA, acetyl-CoA; ANT, adenine nucleotide translocator; CK, creatine kinase; CPT I, carnitine palmitoyltransferase I; Cr, creatine; ER, endoplasmic reticulum; ETC, electron transport chain; FA, fatty acid; FA-CoA, acyl-CoA; FAT, fatty acid transporters; G6P, glucose 6-phosphate; GLUT, glucose transporter; PDH, pyruvate dehydrogenase; PGC-1α, peroxisome proliferator-activated receptor gamma coactivater-1α; PPARα, peroxisome proliferator-activated receptor alpha; ROS, reactive oxygen species.

Doxorubicin is a chemotherapeutic antibiotic of the anthracycline group first isolated in 1969 from *Streptomyces peucetius* [8]. It is regularly used to treat solid and haematological malignancies, including breast cancer, ovarian cancer and acute myeloid leukaemia [9]. Though efficacious, doxorubicin usage clinically is restricted by its cardiac side-effects. Though doxorubicin is associated with a number of acute cardiovascular complications, these are usually minor and transient [10]. However, longer term CTIC is a serious complication of treatment, with doxorubicin-induced heart failure (HF) carrying a mortality rate of 60% once diagnosed [11]. The total dose of doxorubicin is a major risk factor in CTIC development [11,12,13], with an incidence of 3–5% at 400 mg/m^2^ rising to 18–48% at a 700 mg/m^2^ cumulative dose [11,14]. Currently, the primary defence against doxorubicin-HF is limiting the cumulative dose to less than 450 mg/m^2^, but this also limits the therapeutic benefit and does not prevent all cases of CTIC [15]. There are several proposed mechanisms to account for doxorubicin cardiotoxicity, likely distinct from its anti-tumour inhibition of topoisomerase II causing disruption of DNA synthesis [15]. Oxidative stress [16], altered cardiac gene expression [15] and mitochondrial dysfunction [17] have been suggested as some mechanisms of doxorubicin-induced cardiotoxicity [18,19]. Indeed cardiomyocyte death may be a multifactorial process [20] (Figure 1). 

The oxidative stress hypothesis remains the most popular theory of cardiotoxicity and has been reviewed extensively elsewhere [16]. As doxorubicin contains a quinone moiety, it can undergo redox cycling, resulting in the production of reactive oxygen species (ROS) and placing oxidative stress upon the cell. This leads to numerous downstream effects such as mitochondrial dysfunction, inflammation and apoptotic pathway activation [16]. The mitochondrion is hypothesised to be both a source and a target of ROS, with subsequent mitochondrial dysfunction associated with CTIC. As oxidative phosphorylation within the mitochondrion is responsible for more than 95% of the ATP used within normal cardiomyocytes [4], this dysfunction is hypothesised to alter cellular energetics. Indeed, doxorubicin has been associated with a decrease in the oxidative phosphorylation capacity of mitochondria and with changes to substrate usage and energy transfer, which may be responsible for the energetic failure and death of cardiomyocytes [21]. Redox cycling by doxorubicin has been linked to inhibition of complex I of the electron transport chain (ETC), with further direct inhibition of succinate dehydrogenase, succinate oxidase and cytochrome C oxidase due to doxorubicin’s affinity for cardiolipin, an inner mitochondrial membrane phospholipid [22] (Figure 1). Recently, in silico modelling has proposed that a dysfunctional positive feedback loop develops in the mitochondrion, where ROS production inhibits the ETC, both directly and through damage of mitochondrial DNA, resulting in the production of further ROS and increasing mitochondrial dysfunction until bioenergetic failure occurs [23]. However, there is conflicting evidence regarding the oxidative stress hypothesis, with anti-oxidant therapies providing partial efficacy at best [16] and it has been suggested that oxidative stress may not be responsible for mitochondrial dysfunction and loss, nor any downstream metabolic effects of doxorubicin [24]. However, human-induced pluripotent stem cell (iPSC)-derived mesenchymal stem cell (MSC) administration to murine models of doxorubicin cardiotoxicity demonstrated an attenuation of CTIC through functional mitochondrial transfer to cardiac tissue [25], demonstrating the vital role of mitochondria in the development of CTIC.

Doxorubicin has been hypothesised to directly affect cardiac substrate usage. In particular, radiotracer and metabolomics studies in rats have suggested a decrease in both glucose and fatty acid utilisation preceding the onset of cardiac functional decline [26,27]. Some rat studies have suggested doxorubicin inhibits fatty acid oxidation by preventing mitochondrial entry of long-chain fatty acids through direct inhibition of carnitine palmitoyltransferase I (CPT I) [28], whilst others have suggested this to be due to transcriptional alterations in the fatty acid oxidation pathway within mitochondria [29]. Furthermore, altered cellular gene expression has been hypothesised to be the root cause, as doxorubicin has been found to decrease cardiac expression of peroxisome proliferator-activated receptor alpha (PPARα) in murine models, a key regulator of β-oxidation [30]. In addition, doxorubicin was found to also inhibit the expression of peroxisome proliferator-activated receptor gamma coactivator 1-alpha (PGC-1α), potentially favouring a glycolytic state over fatty acid oxidation, and indicating a loss of mitochondrial biogenesis [30]. A loss of fatty acid oxidation capacity is a pathological event also seen in ischaemic heart disease [4] and may be key to CTIC.

However, the bioenergetic failure of cardiomyocytes may also be partly driven by a loss of cellular energetic buffering and availability of cytoplasmic ATP. In models of cardiotoxicity, doxorubicin has been shown to inhibit the adenine nucleotide translocator (ANT), which is responsible for exchanging free ATP for ADP across the mitochondrial inner membrane, compromising the availability of cytosolic ATP, despite mitochondrial production [30]. Moreover, doxorubicin has been shown to impair the creatine kinase reaction in cardiomyocytes [31]. Creatine kinase is the key enzyme which facilitates the reaction converting creatine to phosphocreatine (using a phosphate group from ATP), providing a vital rapid energy buffer. The reaction is reversed during high energy usage, providing an immediate source of ATP—the loss of this system constitutes the loss of an important metabolic safety net [31]. In vivo animal magnetic resonance spectroscopy studies have shown that there is a decrease in the phosphocreatine:ATP ratio early on with doxorubicin administration, prior to cardiac decline, suggesting the loss of high-energy phosphate buffering could constitute an important role in energetic failure of the cell [32] (Figure 1).

More recently, perturbed AMP-activated protein kinase (AMPK) signalling has been proposed as another potential mechanism of doxorubicin cardiotoxicity [33]. AMPK is a heterotrimeric protein complex, with a catalytic α subunit and regulatory β and γ subunits [34]. AMPK is an important regulator of cellular energy homeostasis due to AMP allosterically activating the γ subunit [35]. Further activation occurs at phosphorylation sites on both the α and β subunits, through the action of AMPK kinases [36,37]. Once activated, cardiac AMPK phosphorylates several downstream effectors, such as phosphofructokinase-2 (PFK2) and glucose transporter 4 (GLUT4), resulting in increased glucose uptake and metabolism. Similarly, fatty acid uptake and metabolism is also upregulated both directly and indirectly by AMPK [37]. AMPK activation furthermore promotes mitochondrial biogenesis through increased expression of genes such as PGC-1α [38]. As well as upregulating ATP production, AMPK downregulates anabolic pathways such as protein synthesis and cellular proliferation, primarily thought to be via inhibition of the mechanistic target of rapamycin (mTOR) [33]. Doxorubicin has repeatedly been shown to inhibit cardiac AMPK in a variety of in vivo experimental models, though the precise mechanism of inhibition remains unclear [33,39], and AMPK’s role in doxorubicin cardiotoxicity has been reviewed previously [36]. Therefore, aberrant AMPK inhibition may result in a loss of normal cellular energy signalling and impaired mitochondrial biogenesis, leading to the altered metabolic state seen in doxorubicin cardiotoxicity (Figure 1). Together, these theories all place metabolic alterations centrally in the pathogenesis of doxorubicin cardiomyopathy (Table 1).

### 2.2. Trastuzumab

Trastuzumab is a monoclonal antibody that targets the extracellular region of the human epidermal growth factor receptor 2 (HER2), causing inhibition of the pathway. HER2, an oncogene, is overexpressed in around 20–30% of breast cancers. Therefore, the use of trastuzumab adjuvant oncotherapy has proved clinically successful for treatment of these cancers [50]. However, trastuzumab therapy is associated with cardiotoxicity in 20–45% of patients [51,52,53] and heart failure in 2–3% of patients [51,52]. Though some of this damage is thought to be reversible upon trastuzumab cessation, there is a clear element of long-term, irreversible damage to the myocardium [54], and cardiotoxicity is strongly associated with treatment interruption [53], highlighting the severity of the issue. Moreover, trastuzumab is often used as an adjuvant therapy with doxorubicin, further potentiating cardiotoxicity [54]. Mechanisms underlying trastuzumab’s cardiotoxic effect are thought to be linked to the direct inhibition of the HER2 pathway, which is expressed in adult human cardiomyocytes [55]. Metabolically, these include the production of ROS, altered gene expression, and changes to ATP production [7,56]. This metabolic angle shall be explored here (Table 1).

Mitochondrial dysfunction may lead to cardiomyocyte death in trastuzumab CTIC. Recently, mitochondrial dysfunction has been demonstrated in rabbits administered subcutaneous trastuzumab, with disorganised cristae and disruption to both the inner and outer membranes of the mitochondria evident. Oral antioxidant administration was able to prevent some of these effects, suggesting that ROS at the level of the mitochondrion may play a role in dysfunction, though this purely descriptive study is limited in nature [41]. Supporting this, in vitro studies have found that erbB2 (the non-human equivalent of HER2) blockade increases ROS production, likely at the site of the mitochondrion, which may lead to cell death [57]. Indeed, erbB2 has been shown to translocate to the mitochondria and regulate cellular metabolism in other cell lines [58]. Together, one could suggest that trastuzumab’s direct inhibition of HER2 may lead to pathological alterations in mitochondrial function, creating energetic deficits alongside ROS production.

Trastuzumab may also act upon AMPK, the master cellular energy regulator, to place cardiomyocytes in a pathological energetic state. In embryonic human primary cardiomyocytes, it was first demonstrated that trastuzumab was unable to activate AMPK, though another HER2 inhibitor, GW2974, was able to activate AMPK. AMPK activation was demonstrated to stimulate fatty acid oxidation, providing a metabolic stress response deemed protective to cardiomyocytes in the face of a TNFα challenge [59]. Recently, two studies in human induced pluripotent stem cell-derived cardiomyocytes (iPSC-CMs) have shown trastuzumab to dysregulate metabolism [42,43], likely at the level of AMPK [42]. iPSC-CMs treated with trastuzumab demonstrate a marked contractile dysfunction, mimicking the clinical picture of CTIC. iPSC-CMs treated with trastuzumab were shown to have lower levels of AMPK phosphorylation (corresponding to activation), glucose uptake, cellular ATP, oxidative phosphorylation complexes and mitochondrial respiratory capacity [42,43], indicating a widespread energetic impairment. Transcriptome pathway analysis confirmed significant alterations in the expression of genes relating to metabolic pathways [42,43]. In this model, AICAR (an AMPK activator), alongside AMPK potentiators such as metformin, rosiglitazone, resveratrol and lipoic acid were able to avert these defective metabolic effects and showed a rescue of cardiotoxic symptoms and cardiomyocyte contraction [42], suggesting potential promises in treatment centred on AMPK modulation. Thus, AMPK inhibition may lie at the heart of trastuzumab-related cardiotoxicity, resulting in metabolic alterations which lead to cellular death (Figure 1).

### 2.3. Sunitinib

Sunitinib is a small-molecule, multi-targeted receptor tyrosine kinase inhibitor, which inhibits a wide variety of targets including platelet-derived growth factor receptors (PDGFRs) and vascular endothelial growth factor receptors (VEGFRs) amongst others. It is used in the treatment of advanced renal cell carcinoma, gastrointestinal stromal tumours, pancreatic cancer and neuroendocrine tumours [60]. As with other tyrosine kinase inhibitors, sunitinib is associated with a severe risk of cardiotoxicity, affecting up to 30% of patients, with severe cardiovascular events seen in up to 10% [61]. Approximately half of this cardiac dysfunction is thought to be reversible, leaving a significant cardiovascular burden of risk associated with the drug [62].

The mechanisms underlying sunitinib cardiotoxicity remain relatively unexplored, though it is thought to include coronary microvascular dysfunction due to effects on VEGFR and PDGFR [60,63]. However, off-target inhibition of AMPK has also been suggested as a key element in toxicity. One such study found that sunitinib causes a loss of the mitochondrial membrane potential and bioenergetic failure within cardiomyocytes. In mice, sunitinib was found to reduce the activity of cardiac AMPK, thought to be due to direct inhibition. Indeed, adenoviral transfer of a constitutively active form of AMPK decreased sunitinib induced cell death, suggesting AMPK to be a key target in sunitinib cardiotoxicity [45]. In agreement, a recent study in a mouse model found that trimetazidine (an anti-angina drug) was able to ameliorate sunitinib CTIC through AMPK activation [46] (Table 1). Overall, sunitinib may in part cause a bioenergetic failure within cardiomyocytes through inhibition of AMPK, amongst other effects (Figure 1).

### 2.4. Imatinib

Imatinib is a small molecule inhibitor of the constitutively active fusion BCR-ABL kinase which is present in more than 90% of chronic myeloid leukaemia (CML) cases and 30% of B-cell acute lymphoblastic leukaemia cases [64]. Imatinib’s effective inhibition has revolutionised the treatment of these diseases, but concerns have been raised about cardiotoxicity associated with usage [65]. Despite initial fears, CTIC is thought to be a rare event with imatinib, with just eight cases of heart failure attributed to imatinib in a retrospective analysis of 1276 patients [66]. Nevertheless, an understanding of cardiotoxicity may prove important for the treatment of these rare cases and provide insight into the mechanism of similar cardiotoxic drugs. Initial investigations in neonatal rat ventricular cardiomyocytes have suggested imatinib may induce stress within the endoplasmic reticulum, leading to mitochondrial failure and cellular death [65]. However, a more recent study on H9c [2] cells has suggested oxidative stress to be the initial factor leading to mitochondrial dysfunction and eventual apoptosis, dependent on the mitochondrial scaffold protein Sab [67]. Whilst far from conclusive, mitochondrial dysfunction may play a role in imatinib-mediated cardiotoxicity (Figure 1).

### 2.5. Ponatinib

Ponatinib is a third generation BCR-ABL kinase inhibitor which was created to counter the T315I gatekeeper mutation which causes imatinib resistance in patients with CML [68]. Despite being clinically efficacious, ponatinib has been associated with a high rate of cardiovascular adverse events, with a recent retrospective study finding 5% of patients with HF and more than 20% to experience an adverse cardiovascular event with ponatinib therapy [68]. Analysis of the FDA adverse event reporting system database has found ponatinib to carry the greatest risk of cardiotoxicity amongst all kinase inhibitors [69], with disproportionally high reporting of cardiac failure, ischaemic heart disease and hypertension compared to other anticancer therapies for CML [70]. 

Ponatinib’s cardiotoxic effects may be due to both vascular effects on the coronary microvasculature and direct effects on the myocardium itself, linked to the promiscuity of its kinase inhibition [71]. Indeed, ponatinib has been found to be antiangiogenic in vitro [72], and mouse studies have found some evidence of a global microvascular angiopathy in the coronary vessels [73]. In cardiomyocytes, ponatinib has been shown to inhibit the AKT and ERK signalling pathways, inducing cardiomyocyte apoptosis, which can be ameliorated by activation of the pathways with neuregulin-1β [74]. The PI3K-AKT signalling pathway is involved in many downstream effects on cellular metabolism, including glucose uptake and glycolysis. An inhibition of the pathway may negatively affect the cellular energetic state, favouring cardiomyocyte apoptosis [75]. Indeed, ponatinib has been shown to strongly impair oxidative metabolism and weakly impair glycolysis in vitro within hepatocytes, albeit at higher concentrations that are reached in plasma, though potentially at those seen in the heart/liver [76]. 

### 2.6. Radiotherapy

Radiotherapy is an important treatment option as either a monotherapy or adjuvant for a wide variety of cancers. However, adverse cardiovascular events, labelled under radiation-induced heart disease (RIHD), have caused significant morbidity and mortality in patients. Though the incidence varies depending on the specific malignancy and dose of radiotherapy used, RIHD has been widely reported in Hodgkin’s lymphoma [77] and breast cancer [78,79]. In a recent study examining breast cancer patients, the cumulative incidence of cardiac events was found to increase proportionally by 16.5% per Gray of radiation patients were exposed to [79]. 

Endothelial injury, inflammation, oxidative stress and ER stress have all been implicated in the mechanism of RIHD [80]. Despite endothelial cell injury gaining prominence as the primary cause of RIHD [80], clinical radiotracer studies have shown myocardial alterations in metabolic uptake evident in irradiated segments of the heart, which was not linked to the vascular territories of coronary arteries [48]. Further, a recent in vivo study in beagles has suggested that much of the sequalae of RIHD is not associated with an increase in pro-inflammatory cytokines. Instead, the team identified an increase in GLUT4 expression and a decrease in CPT1 expression in irradiated myocardium, alongside mitochondrial damage and increased glucose uptake, suggesting a switch from fatty acid oxidation to glycolysis [47] (Figure 1), a metabolic shift seen in the pathogenesis of ischaemic heart failure [4]. These findings extend upon a clinical study of patients with oesophageal cancer, who were treated with chemoradiotherapy. It was found that 20% of these patients exhibited increased glucose uptake (through ^18^F-FDG PET-metabolic imaging) in the irradiated myocardium, though this was not linked to any RIHD, and the adjuvant chemotherapy presents a significant confounding variable [49]. Nevertheless, taken together, these studies suggest that myocardial metabolic alterations may play an important role in the pathogenesis of RIHD.

## 3. Predicting Cardiotoxicity Using Metabolic Markers

Though our understanding of the mechanisms behind CTIC is improving, clinically a major unmet need is to be able to predict which patients are likely to suffer from CTIC. The alterations in myocardial metabolism associated with CTIC present a potential solution to this issue. As myocardial metabolism is within the pathophysiology of disease, early identification of metabolic shifts could be predictive of cardiotoxicity and allow for appropriate alterations to therapy to be taken. There are a variety of techniques which can be conducted to do this, including measuring blood biomarkers, radiolabelled PET-CT imaging and hyperpolarised MR imaging (Figure 1). Here, we shall examine how these techniques have been applied to identify early signs of CTIC, as well as a wider exploration of their potential use based on studies from other conditions (human studies summarised in Table 2).

### 3.1. Blood Biomarkers

Blood biomarkers are easily obtainable and tested for, and if found to provide a high sensitivity and specificity, could be rapidly put into clinical practice. Traditional biomarkers of cardiac injury may present too late for predictive therapy, for example, an increase in troponin-I levels after anthracycline administration has been associated with cardiotoxicity, but this is an indication that cardiac injury has already occurred [83]. Instead, an examination of plasma metabolites can provide insight into changes in cellular metabolism, potentially identifying early changes in bioenergetics before the onset of cardiac damage. Recently, this was examined in a small study of patients with HER2+ breast cancer treated with adjuvant therapy of anthracyclines, taxanes and trastuzumab. Using liquid chromatography mass spectroscopy (LC-MS), the team analysed fasting blood plasma samples of patients at various timepoints in therapy. The group found that patients who went on to develop cardiotoxicity exhibited significantly lower levels of citric acid and aconitic acid prior to functional changes detected by echocardiography. Indeed, the magnitude of the decrease of citric acid/aconitic acid was correlated with the change in the left ventricular ejection fraction (LVEF) at a later time point [40]. Though this study did not examine the predictive value of these metabolites, it highlights early changes in the citric acid cycle as a potential source of important plasma biomarkers for cardiotoxicity.

Though there are few studies directly looking at CTIC, other heart failure studies suggest that plasma biomarkers of metabolism could prove promising. In one such recent approach, baseline plasma samples were taken from patients in a randomised controlled trial examining the role of spironolactone in heart failure with preserved ejection fraction. Elevated levels of fatty acid binding protein-4, a protein correlated to the rate of fatty acid metabolism, were shown to be strongly linked to the later development of heart failure, independent of other risk factors [84]. This highlights how baseline plasma studies could provide a very early prediction of cardiac toxicity if applied to CTIC.

However, understanding the meaning of these plasma biomarker studies can be difficult as they may not directly correlate to cardiac metabolism. In a recent study of heart failure patients, a metabolomics approach was used, taking blood samples from the femoral artery, coronary sinus and femoral vein in an attempt to directly assess cardiac metabolism. Heart failure was shown to increase ketone body and lactate usage within the heart itself, but this was only apparent through a comparison of arterial and coronary sinus samples, not femoral vein samples, demonstrating that peripheral blood samples may not be representative of the picture in the heart [85]. Nevertheless, advances in biomarker-based techniques could rapidly revolutionise our predictive capabilities in cardiotoxicity.

### 3.2. ^12^F-FDG PET/CT

Cancer therapy-induced metabolic changes in part can alter substrate use within the myocardium. The use of radiotracers for metabolic substrate uptake, combined with anatomical imaging, could provide a valuable technique to visualise changes in cardiac glucose uptake, which could predict future changes to cardiac function. ^18^F-fluorodeoxyglucose (^18^F-FDG) is a radiolabelled analogue of glucose, which is taken up by cellular glucose transporters and is phosphorylated by hexokinases, where it becomes trapped within the cell and cannot undergo further metabolism. This accumulation can be seen using positron emission tomography (PET) alongside computerised tomography (CT) for signal co-localisation with tissue anatomy [86]. ^18^F-FDG PET/CT is a technique widely used to identify metabolically active cancers, but also has potential to detect alterations to glucose uptake in the myocardium.

A recent animal model of RIHD has suggested ^18^F-FDG PET/CT can be used to identify metabolic changes prior to any detectable changes in cardiac function. Cardiac ^18^F-FDG standardised uptake values were found to be significantly increased in beagles who had undergone myocardial irradiation, compared to controls, prior to any functional changes being seen, suggesting this may be a valuable technique for the early detection of CTIC [47] and highlighting a shift towards glucose usage. However, studies in humans have been less clear. A small study of lymphoma patients treated with doxorubicin found it to have mixed effects upon ^18^F-FDG uptake, though cardiac function parameters were unavailable [81]. On the other hand, a recent retrospective study of breast cancer patients who had anthracycline- or trastuzumab-based oncotherapy found an association between ^18^F-FDG uptake in the right ventricular myocardium immediately post-therapy and the later development of cardiotoxicity after controlling for age, radiotherapy and treatment type. Though not early enough to change treatment choices, this may still be of prognostic value [44]. However, these results must be treated with caution—these retrospective ^18^F-FDG PET/CT scans are conducted primarily for tumour visualisation, with a differing protocol to myocardial specific ^18^F-FDG PET/CT, which may lead to spurious results. Nevertheless, the technique shows some promise and warrants further clinical exploration. As such, a case-control trial is currently recruiting lymphoma patients to assess the value of ^18^F-FDG PET/CT as a predictive tool for chemo- or immunotherapy induced cardiotoxicity (NCT04555642) [87].

### 3.3. Hyperpolarised ^13^C MRI

Though ^18^F-FDG PET/CT has been proven to be a useful imaging technique, it is limited to demonstrating the cellular uptake of glucose and cannot show any alterations to the metabolic fate of substrates. ^13^C magnetic resonance spectroscopy allows for the direct evaluation of the metabolic fates of the ^13^C-containing compound. The low sensitivity of this technique has been addressed by hyperpolarised magnetic resonance, which increases the ^13^C signal by several orders of magnitude [88]. This makes the technique viable for imaging metabolic fluxes in patients, and hyperpolarised ^13^C MRI is well-tolerated by patients [89]. The technique has been successfully used in a porcine model of heart failure, demonstrating a decrease in pyruvate dehydrogenase flux (representing a move to anaerobic glycolysis) prior to any functional changes seen using echocardiography [90]. Further, the technique has been used to demonstrate cardiac metabolic alterations in diabetic patients [91].

Looking at CTIC, there are several studies that support the use of hyperpolarised ^13^C MRI to predict functional decline. In a clinically relevant rat model of doxorubicin-induced cardiotoxicity, one such study utilised hyperpolarised MRI with [1-^13^C]pyruvate and [2-^13^C]pyruvate to assess changes to pyruvate dehydrogenase flux and TCA cycle flux, respectively. Doxorubicin administration was associated with a decrease in the [^13^C]bicarbonate:[^13^C] pyruvate ratio compared to controls prior to the detection of cardiac dysfunction. This reflects a decreased flux through pyruvate dehydrogenase, in line with expected decreases in mitochondrial oxidative phosphorylation [24]. This represents an important detection of an early sign of CTIC. Furthermore, a pilot study in 10 breast cancer patients treated with adjuvant doxorubicin found similar results of a decrease in the [^13^C]bicarbonate/total ^13^C signal post doxorubicin administration using hyperpolarized [1-^13^C]pyruvate [82]. As a result of these promising studies, there are clinical trials currently recruiting breast cancer [92] and thoracic cancer [93] patients to evaluate the feasibility of [1-^13^C]pyruvate hyperpolarised MRI to detect radiation-induced cardiotoxicity (NCT03685175, NCT04044872). Overall, there have been rapid advances in the utility of hyperpolarized ^13^C MRI, and there is great promise for its diagnostic potential, despite its currently limited availability and high cost [94].

## 4. Adjuvant Cardiotherapy

Being able to identify which patients are likely to experience CTIC would allow for early decision making about therapeutic options, not just limited to halting or changing cancer therapies. Instead, this could allow for the early administration of adjuvant cardioprotective therapies, allowing for effective chemotherapy regimens to be continued, whilst protecting the heart of vulnerable patients. Though this is a field in its infancy, as many of the pathological metabolic alterations in CTIC are similar to those seen in heart failure of other origins, we can look to recent developments in ischaemic heart failure to envision some potential cardioprotective therapies which could be applied more widely to CTIC. Here, we explore two potential therapies—the sodium-glucose transport protein 2 (SGLT2) inhibitors, and metformin; both may provide a cardioprotective effect through metabolic alterations, representing a small pool of potential therapies in this rapidly expanding field (Figure 1).

### 4.1. Metformin

Firstly, metformin is a widely used first-line drug in the treatment of type 2 diabetes mellitus, with a strong safety profile. In addition to its glucoregulatory effects, primarily thought to be due to suppression of hepatic gluconeogenesis, metformin is thought to alter energy metabolism in part through an activation of AMPK, though this is a hotly debated topic [95]. As a side effect of therapy, metformin has been shown to be cardioprotective in diabetes, going beyond its role in glucose control [96]. Beyond diabetes, metformin has been demonstrated to be effective at reducing mortality in heart failure patients with preserved ejection fraction in a recent meta-analysis [97]. A cardioprotective effect in heart failure has been hypothesised to act in two ways: by stimulating the insulin-mediated uptake of glucose in the myocardium and by activating AMPK and restoring pathological cardiac energetics [96]. These positive findings all suggest metformin may be a strong candidate as an adjuvant therapy to prevent CTIC.

Indeed, a recent retrospective database study of patients with early stage breast cancer who received adjuvant breast radiotherapy found metformin use to significantly decrease the risk of major cardiac events (HR = 0.79), suggesting metformin could reduce the risk of RIHD [98]. Though a limited retrospective study, it is an early sign that metformin could provide value if given to patients likely to develop CTIC. Looking to preclinical models, metformin has been shown to provide a cardioprotective effect against doxorubicin in rats [99]. In vitro, metformin’s cardioprotection against doxorubicin has been demonstrated to be directly due through AMPK activation, with AMPK inhibitors able to abolish any protective effect [100]. This AMPK activation directly reverses the inhibition seen with doxorubicin administration and may also reduce oxidative stress within the cell through its role as an energy stress sensor [101]. In human iPSCs, metformin has also been shown to ameliorate the trastuzumab-induced dysfunction, put down to its activating effects on AMPK [42]. Interestingly, metformin may play a dual-role in cancer therapy, as it has been associated with a decrease in cancer mortality in patients with type II diabetes [102]. This was recently investigated by the METTEN study, which examined whether metformin was able to increase the pathological complete response (clearance of cancer) in HER2-positive breast cancers treated with traditional chemotherapeutics + trastuzumab [103]. Though the study was inconclusive due to a lack of power, this highlights the research interest into the potential for metformin as a cancer therapy adjuvant. With further research, metformin can be envisioned to potentially both improve cancer therapy responsiveness as well as providing protection against CTIC.

### 4.2. SGLT2 Inhibitors

SGLT2 inhibitors are a modern class of drugs which were originally introduced as a treatment for type 2 diabetes mellitus, as they block glucose reabsorption in the proximal convoluted tubule of the kidney, increasing glycosuria and reducing blood glucose levels [104]. The EMPA-REG OUTCOME trial examined how the SGLT2 inhibitor empagliflozin (EMPA) affected cardiovascular outcomes in type 2 diabetes mellitus patients. It found EMPA to significantly reduce the risk of adverse cardiovascular events, with post-hoc analysis finding the effect to be independent of glycaemic control [105]. Moreover, the DAPA-HF trial showed similar outcomes with another SGLT2 inhibitor, dapagliflozin, in heart failure patients with reduced ejection fraction, regardless of type 2 diabetes mellitus status [106]. This suggests SGLT2 inhibitors may have an important cardioprotective role, which cannot solely be explained through its diuretic effect, suggesting it to be a strong candidate against CTIC.

Indeed, one widely proposed mechanism of cardioprotection with SGLT2 inhbitors has been the ‘thrifty substrate’ hypothesis, suggesting that a decrease in available glucose due to SGLT2 inhibitors forces metabolism to favour the oxidation of free fatty acids and places the body into a ketogenic state, creating a mild, persistant hyperketonemia. The preferential oxidation of ketones within the heart is more oxygen efficient, reducing the stress placed upon the heart [107], and removes a reliance upon anaerobic glycolysis. A similar mechanism may confer cardioprotection against cancer therapies. A number of murine models have found EMPA to prevent doxorubicin induced cardiotoxicity [108,109,110,111,112], with an improvement in LV function that is not seen with pure reductions in circulating volume, such as furosemide treatment [108]. One such recent study attributes this cardioprotection due to elevations in the ketone β-hydroxybutyrate which were seen upon EMPA administration. Direct β-hydroxybutyrate treatment alongside doxorubicin was sufficient to reduce symptoms of cardiotoxicity, suggesting the production of a hyperketonic state may be protective. Within cardiomyocytes, β-hydroxybutyrate administration, alongside doxorubicin, resulted in a decrease in mitochondrial dysfunction and ROS levels [112], recaptiulating effects of ketone administration seen in mouse models of ischaemia-reperfusion [113]. Despite this attractive hypothesis, other studies have suggested a variety of cardioprotective mechanisms of EMPA cardioprotection including upregulated PGC-1α [109], altered autophagy [110] and decreases in pro-inflammatory cytokines [111]. This is an actively expanding field, and there are likely to be rapid further advances on the topic. Though to the best of our knowledge in vivo studies of EMPA cardioprotection with other cancer therapies have not been performed to date, early cardiomyocyte studies with dapagliflozin suggest it may also provide benefit with trastuzumab treatment [114].

### 4.3. Resveratrol

Resveratrol ([3,5],4′-trihydroxy-trans-stilbene) is a naturally occurring polyphenol, found in a number of plants, notably in high concentrations in the skin and seeds of grapes [115]. Available as a dietary supplement, resveratrol has attracted particular interest within the scientific community due its antioxidant effect, usually tested at far higher concentrations than present naturally [115]. Though not yet in clinical use, resveratrol’s potential anti-inflammatory, anti-cancer and antimicrobial effects may provide therapeutic benefit for a variety of disaeses. In the cardiac sphere, a randomised controlled trial of 40 patients demonstrated that resveratrol administration in stable coronary artery disease patients (who had a history of myocardial infarction) significantly impoved LV diastolic function and resulted in a decrease in circulating LDL cholesterol [116]. Previous in vivo studies in murine postinfarction heart failure models have suggested this may be due, at least in part, to an antioxidant effect decreasing oxidative stress. Signalling pathway alterations have also been implicated in the cardioprotective role of resveratrol, including the AKT pathway, which is involved in cellular survival signalling, but also glucose uptake and metabolism control [117]. As a safe, well-tolerated compound, these findings may extend to a potential protective effect against CTIC. 

In the context of CTIC, resveratrol’s role has previously been expertly reviewed [118], with a range of potential cardioprotective effects. Here, we shall focus upon the possible metabolic effects of the drug as a potential cancer therapy adjuvant. In vivo, resveratrol has been demonstrated in murine models to offer cardioprotection against doxorubicin administration [119,120] and may potentiate the anticancer effect of doxorubicin as well [121]. Some of this cardioprotection may be due to a restoration of mitochondrial dysfunction, perhaps due to AMPK activation [122], SIRT1 activation [123], or directly increased expression of mitochondrial ETC complexes [120]. The direct AMPK effect has also been explored in human-iPSC models of trastuzumab CTIC, where resveratrol was able to overcome contractile dysfunction induced by trastuzumab, thought to be due to its role in activating AMPK [42]. Looking to radiotherapy, black grape juice (containing high concentrations of resveratrol) administration to rats was found to prevent an increase in lactate dehydrogenase levels after radiation administration, suggesting it may have a protective effect at the myocardium, though this study is of limited quality [124]. Recently, a metabolomics-based approach analysing cardiac tissue in murine models of RIHD demonstrated that resveratrol can prevent the radiation-induced decrease in choline-containing metabolites and unsaturated fatty acid metabolites, implying alterations to cardiac metabolism may play a role in its cardioprotective effect [125]. Though our understanding of resveratrol is more limited than other potential adjuvant therapies, more research could yield exciting insights into a possible therapeutic role for this compound in CTIC patients. 

## 5. Conclusions

In summary, CTIC represents a severe limitation of cancer therapies, likely in part due to metabolic disturbances at the level of the cardiomyocyte. It appears that both mitochondria and the AMPK signalling pathway may be important mediators of this metabolic dysfunction, both resulting in potential bioenergetic failure of the cardiomyocyte. Despite a need for more clinical research, it appears more novel targeted chemotherapies such as sunitinib and ponatinib may also exert cardiotoxic effects through these pathways. It is evident that changes in myocardial metabolism, preceding functional changes in CTIC can be detected through a variety of techniques, including plasma metabolites (biomarkers), ^18^F-FDG PET/CT and hyperpolarised ^13^C MRI, with rapid advances in diagnostic potential. A refinement of these techniques will make them permissible for routine clinical use, allowing for the detection and prediction of patients susceptible to CTIC, at an early enough stage to prevent functional decline from occurring. An innovative approach to drugs which can alter metabolism, applied in this context, could provide a cardioprotective effect as an adjuvant therapy in targeted vulnerable patients. Though more translational research is needed, exploring myocardial metabolism for early diagnosis and treatment of CTIC is an exciting and emerging avenue.

## Figures and Tables

**Table 1:** Cardiac metabolic alterations of cancer therapies implicated in the pathogenesis of cardiotoxicity. In vivo and iPSC studies have been selected for clinically relevant mechanistic insight. AMPK, AMP-activated protein kinase; CPT I, carnitine palmitoyltransferase I; GLUT4, glucose transporter type 4; mTOR, mechanistic target of rapamycin; PCr, phosphocreatine; PGC-1α, peroxisome proliferator-activated receptor gamma coactivator 1-alpha; PPARα, peroxisome proliferator-activated receptor alpha.

Model of Disease	Metabolic Alteration	Ref.
Doxorubicin		
Rat	Decrease in long-chain fatty acid oxidation Inhibition of CPT I	[28]
Rat	Decrease in long-chain fatty acid oxidation	[29]
Rat	Abnormal amino acid metabolism Abnormal fatty acid metabolism Abnormal glycerol phospholipid metabolism	[27]
Mouse	Decrease in expression of PPARα Decrease in expression of PGC-1α Inhibition of adenine nucleotide translocator Decrease in availability of cytosolic ATP	[30]
Mouse	Decrease in PCr to ATP ratio Loss of high energy phosphate buffering	[32]
Rat	Inhibition of AMPK Inhibition of acetyl-CoA carboxylase	[33]
Rat	Inhibition of carbohydrate oxidation Decrease in mitochondrial number	[24]
Human	Alterations in citric acid and aconitic acid levels Alterations in purine and pyrimidine metabolism	[40]
**Trastuzumab**		
Rabbit	Mitochondrial dysfunction, likely due to ROS	[41]
Cardiac iPSCs	Inhibition of AMPK Decrease in cellular ATP Alterations in mTOR signalling pathway Decrease in glucose uptake	[42]
Cardiac iPSCs	Decrease in expression of small molecule metabolism genes Decrease in expression of cholesterol and sterol processing genes Decrease in glucose uptake	[43]
Human	Increase in glucose uptake	[44]
**Sunitinib**		
Mouse	Inhibition of AMPK Loss of mitochondrial membrane potential	[45]
Mouse	Inhibition of AMPK Inhibition of mTOR	[46]
**Imatinib**		
Lack of clinically relevant studies		
**Ponatinib**		
Lack of clinically relevant studies		
**Radiotherapy**		
Beagle	Increase in glucose uptake Increase in GLUT4 expression Decrease in CPT I expression	[47]
Human	Increase in glucose uptake	[48]
Human	Increase in glucose uptake	[49]

**Table 2:** Clinical studies investigating the correlation of plasma metabolites and metabolic imaging with the onset of cardiotoxicity. ^18^F-FDG PET/CT, ^18^F-fluorodeoxyglucose positron emission tomography-computed tomography; HER2, human epidermal growth factor receptor 2; LC-MS, liquid chromatography–mass spectrometry; SUV, standardized uptake value.

Patient Population	Type of Study	Cancer Therapy	Sample Size	Findings	Ref.
Blood Biomarkers					
HER2+ Breast Cancer	Case-control	Anthracyclines, Taxanes, Trastuzumab	38	LC-MS metabolomics Heart failure preceded by: ● Decreased citric acid:isocitric acid ratio ● Altered purine acid & pyrimidine metabolites	[40]
**^18^F-FDG PET/CT**					
Breast Cancer	Retrospective Logistic Regression	Anthracyclines, Trastuzumab	121	SUV in right ventricular wall post therapy associated with development of cardiotoxicity	[44]
Lymphoma	Retrospective Analysis	Doxorubicin	18	Unclear alterations in ^18^F-FDG uptake	[81]
**Hyperpolarised ^13^C MRI**					
Breast Cancer	Prospective	Doxorubicin	9	Early decline in [^13^C]bicarbonate/total ^13^C signal	[82]
